# Near-zero-index ultra-fast pulse characterization

**DOI:** 10.1038/s41467-022-31151-4

**Published:** 2022-06-20

**Authors:** Wallace Jaffray, Federico Belli, Enrico G. Carnemolla, Catalina Dobas, Mark Mackenzie, John Travers, Ajoy K. Kar, Matteo Clerici, Clayton DeVault, Vladimir M. Shalaev, Alexandra Boltasseva, Marcello Ferrera

**Affiliations:** 1 Institute of Photonics and Quantum Sciences, Heriot-Watt University, SUPA, Edinburgh, EH14 4AS UK; 2 James Watt School of Engineering, University of Glasgow, Glasgow, G12 8QQ UK; 3 School of Engineering and Applied Sciences, Harvard University, Cambridge, MA 02138 USA; 4 School of Electrical and Computer Engineering and Birck Nanotechnology Center, Purdue University, West Lafayette, IN 47907 USA

**Keywords:** Nonlinear optics, Ultrafast photonics

## Abstract

Transparent conducting oxides exhibit giant optical nonlinearities in the near-infrared window where their linear index approaches zero. Despite the magnitude and speed of these nonlinearities, a “killer” optical application for these compounds has yet to be found. Because of the absorptive nature of the typically used intraband transitions, out-of-plane configurations with short optical paths should be considered. In this direction, we propose an alternative frequency-resolved optical gating scheme for the characterization of ultra-fast optical pulses that exploits near-zero-index aluminium zinc oxide thin films. Besides the technological advantages in terms of manufacturability and cost, our system outperforms commercial modules in key metrics, such as operational bandwidth, sensitivity, and robustness. The performance enhancement comes with the additional benefit of simultaneous self-phase-matched second and third harmonic generation. Because of the fundamental importance of novel methodologies to characterise ultra-fast events, our solution could be of fundamental use for numerous research labs and industries.

## Abstract (web summary)

Frequency resolved optical gating is the core method for characterising ultra-fast optical pulses. Here, the authors use zero-index nonlinearities to largely enhance key performances and enable simultaneous second and third harmonic measurements.

## Introduction

For over two decades, Transparent Conducting Oxides (TCOs) have been largely employed by manufacturers all over the world for the fabrication of photovoltaic systems and touchscreen devices^1^. More recently, it has been discovered that these hybrid materials possess exceptional nonlinear properties when interacting with electromagnetic radiation within the so-called near-zero-index (NZI) window^2,3^, where the material’s refractive index approaches zero. The origins of this remarkable nonlinear enhancement are still at the centre of an intense debate within the photonics community. However, it is understood that a slow-light effect, directly linked to the NZI nature of these compounds and their characteristic optical dispersion, is mainly responsible for their extraordinary nonlinearities^4–7^. Numerous experimental demonstrations have been conducted that show the unprecedented nonlinear properties of NZI TCOs^8–14^. Including, but not limited to, an optically induced unitary index change^15^ and an ultra-broad nonlinear frequency shift^4,16,17^.

By referring to the nonlinear Figure of Merit (FoM) for these materials as ${n}_{2}/\alpha$ (where ${n}_{2}$ is the nonlinear Kerr coefficient and $\alpha$ represents linear propagation losses), a comparison can be made between aluminium zinc oxide (AZO), as representative of TCOs, and silicon nitride, a cornerstone material for nonlinear integrated photonic applications. A straightforward evaluation from experimental results leads to FoM_SiN_ = 9.8 × 10^−16^ cm^3^/W, FoM_AZO_ = 1.4 × 10^−16^ cm^3^/W^11,18,19^. From these numbers, it becomes immediately apparent that NZI TCOs are among the most promising candidate materials for designing novel all-optical integrated photonics modules^20^.

Among the most noticeable proof-of-concept applications, which make use of TCOs’ enhanced nonlinearities, are tuneable nanocavities^21^, three-state logic^22^, ultra-fast signal routing^23,24^, polarisation selectors^25^, and nano modulators^26–28^. However, despite all these remarkable experimental demonstrations, the integrated photonics community is still seeking the perfect “killer” application that could take full advantage of TCOs’ unique optical properties. When considering both optical absorption and nonlinearities, it becomes evident that TCOs can give their best performance when sub-micron propagation distances are required. For all these reasons, flat optical devices operating in an out-of-plane geometry are a very attractive option. More specifically, among all the possible devices that meet the previously listed requirements, the clear choice falls on a widespread system for the characterization of ultra-fast optical pulses, namely, the frequency resolved optical gating (FROG) system. This is a very powerful tool widely used in many photonic labs, which can take direct advantage of the fundamental optical nonlinearities of TCO-based ultra-thin films without the need for lithographic patterning. For the sake of completeness, we wish to underline that along the last three decades of intense research in ultra-fast physics, many different alternatives to the FROG system have been developed, including Spectral Phase Interferometry for Direct Electric-field Reconstruction (SPIDER)^29^ and Multiphoton Intrapulse Interference Phase Scan (MIIPS)^30^. Importantly, SPIDER utilises sum-frequency generation, and MIIPS uses second harmonic generation, both of which could benefit from NZI enhanced nonlinearities.

In this work we show how system performance improves when the typical bulk nonlinear crystal in a standard FROG is replaced by an ultra-thin TCO film operating in its NZI region. We demonstrate that, besides registering remarkable improvements in all fundamental metrics (e.g., bandwidth, sensitivity, cost, ease of fabrication, relaxed phase matching requirements, reduced time smearing and dispersion, etc.), NZI films also enable new measurement capabilities stemming from the simultaneous generation of multiple harmonics from a single pulse.

## Results and discussion

### Pulse characterisation background

As previously mentioned, a multitude of pulse characterisation techniques have been developed throughout the history of ultra-fast optics^31,32^. Most of them can be recognised as advancements upon the intensity autocorrelation, which requires nonlinearity to function. In the interest of brevity, we shall focus only on the most relevant autocorrelation acquisition methods, which are based on second harmonic generation (SHG) and third harmonic generation (THG).

To perform an intensity autocorrelation the pulse to be studied is separated by a 50:50 intensity splitter which sends two pulse replicas along different propagation paths, one of which is variable in length (via a delay line). Both pulses are then focused onto a nonlinear medium while spatially and temporally overlapped. By recording the intensity of the nonlinear harmonic signals as a function of the time delay, the autocorrelation is retrieved. This measurement can be framed mathematically as a one-dimensional phase-retrieval problem, to which there is no unique solution, consequently limiting the autocorrelation’s usefulness in the recovery of a pulse’s full temporal amplitude. If we wish to recover the complete phase and amplitude time characteristics of complex pulses, we require a method that provides additional information. A FROG system does precisely this. In a FROG configuration, the entire spectrum of the nonlinear signal is recorded at each time delay, and through an iterative approach, we can recover a solution to this reformatted problem. This solution is almost unique if we know the input spectrum^33^.

### Experimental setup and NZI films

**Fig. 1:**
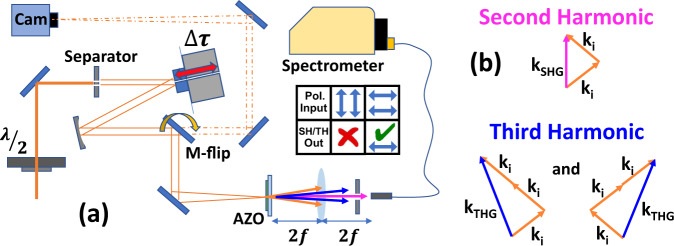
Zero-Index FROG. **a** Experimental setup used to record FROG traces. Central panel summarises input/outputpolarisation states. Maximum frequency conversion was attained for horizontally polarised input beams for both SH and TH (both horizontally polarised). No harmonic generation can be found for a vertically polarised input. **b** Breakdown of phase matching conditions for each harmonic generated in the film.

The specific TCO we focus our attention on is AZO, which does not make use of rare elements, is highly sustainable^34^, possesses a wide statically tuneable NZI window (1200–2000 nm)^35^, and can be fabricated at low temperatures by various CMOS compatible techniques on a huge variety of available substrates^36,37^. Most importantly, AZO is transparent in the visible spectral window, allowing for unattenuated generation of visible light via nonlinear processes^38^. Our FROG system employs a 270 nm film of AZO deposited on a fused silica substrate as the nonlinear element (See Fig. 1a for a detailed layout of the experimental setup). This AZO film stands in for the nonlinear crystal usually found at the core of a FROG, allowing for a number of key improvements compared to an equivalent system employing bulk nonlinearities. By operating in the NZI frequency window and exploiting the consequent enhancement of harmonic generation in AZO, it is possible to acquire SH-and TH-FROG traces simultaneously (see Fig. 1b for details of SH and TH phase matching in our setup).

Interestingly, the recorded SH signal results from the broken inversion symmetry at the film’s surface, but there is no bulk effect due to the centrosymmetry of AZO. Intense surface SH signals have also been recorded in other slow-light devices such as SiN micro-ring resonators^39^. However, our approach allows for higher efficiency and superior directionality without the stringent fabrication requirements linked to the realisation of a high-Q cavity. Furthermore, both harmonics emerging from our thin films are powerful enough to be clearly seen by the naked eye and are spatially separated due to momentum conservation. The latter point allows for the straightforward spatial filtering of cross-pulse and single pulse nonlinearities.

Our experiments started by setting the central pulse wavelength at 1300 nm, which is at the peak of our material's NZI nonlinear enhancement^40^. Harmonics were then generated by the overlap between two pulses incident on the nonlinear film (TH contribution from silica substrate was investigated and found to be negligible). The spectra of the SH (central lobe) and TH (side lobes) were then recorded as a function of pulse delay. Maximum frequency conversion was attained for horizontally polarised input beams, which resulted in horizontally polarised output harmonics, and a much-attenuated nonlinear signal was found for a vertically polarised input. A summary of these results is reported in the inset of Fig. 1a. We highlight that the strong polarisation dichroism observed is a consequence of enhancement of the second and third-order nonlinear processes occurring only for the longitudinal component of the electric field^12,41^. This is because the magnitude of the normal component of the electric field within a medium (right after an interface) is proportional to the incident field amplitude and inversely proportional to the permittivity. For the case of near-zero-index (also epsilon-near-zero) films, this effect produces a local field enhancement that has been previously demonstrated in NZI TCOs^8,10^ and can be enhanced further by varying the angle of incidence (when at horizontal polarisation). We tested for this angular enhancement, which provided an optimal point at 30 degrees with a 35% enhancement in the conversion efficiency over normal incidence (see supplementary material appendix A). It is worth noticing that this effect can occur even at normal incidence due to surface irregularities^42^.

Conventionally, SH- and TH-FROG are not integrated into a single system due to the great difficulty of generating both harmonics with a single pulse in any conveniently measurable amount. Our AZO film overcomes this constraint and generates both harmonics while showing the highest TH generation efficiency in flat systems (8.47 × 10^−5^ with a single beam at normal incidence measured with 100 fs pulses from a 1 kHz laser source).

**Fig. 2:**
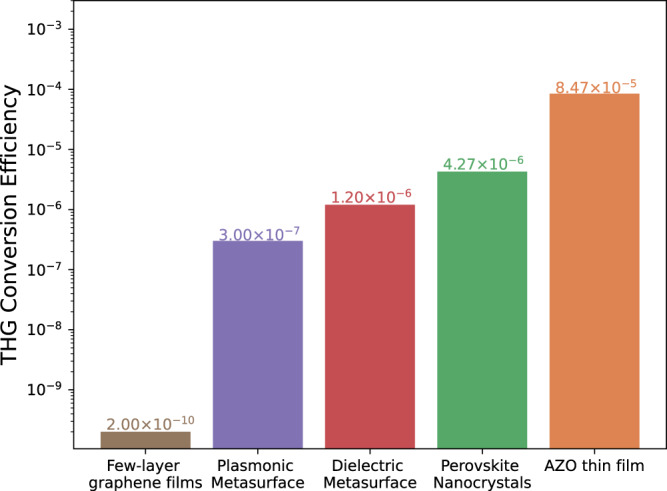
Nonlinear efficiency comparison amongst flat systems. 2D materials, metasurfaces, perovskite nanocrystals, and NZI thin films are considered as benchmark technologies.

Figure 2 and Table 1 compare the efficiency of our AZO film to alternative flat systems using out-of-plane configurations for THG. Representative systems pertaining 2D materials, metasurfaces (both plasmonic and dielectric), emerging semiconductors (perovskites), and TCOs have been considered. Despite the inherent limitations of comparing results from different experimental settings, and the broad variability of the fluences employed in various studies, we believe that the results in Fig. 2 can shine light on the intrinsic advantage of using NZI flat devices for frequency conversion purposes. In fact, from an operational point of view, the optical fluence employed cannot be freely varied in each of the considered systems due to the damage threshold or nonlinear saturation of each material, meta or otherwise. The fact that AZO can be operated at a fluence level on the order of hundreds of mJ/cm^2^ is a benefit in itself, owing to AZO’s high damage threshold that allows for full exploitation of optical nonlinearities. The direct comparison between these alternative flat systems reveals that NZI TCO films enable an unprecedented nonlinear conversion efficiency. It is worth pointing out that the maximum fluence used in our experiments was set at 28 mJ/cm^2^, which is well below the material damage threshold^38,40,43^ and still quite below the nonlinear saturation point.

**Table 1:** Key parameters for THG efficiency.

Method	Reference	Fluence (mJ/cm^2^)	Duration (fs)	THG Efficiency
Few-layer graphene films	^51^	1.00	320	2.0 × 10^−10^
Plasmonic metasurface	^52^	0.02	170	3.0 × 10^−7^
Dielectric metasurface	^53^	0.88	250	1.2 × 10^−6^
Perovskite nanocrystals	^50^	1.50	200	4.27 × 10^−6^
AZO thin film	This work	27.97	100	8.5 × 10^−5^

The table links the frequency conversion efficiencies of different experimentally tested flat systems to both fluence levels and pulse durations.

### NZI FROG measurements

**Fig. 3:**
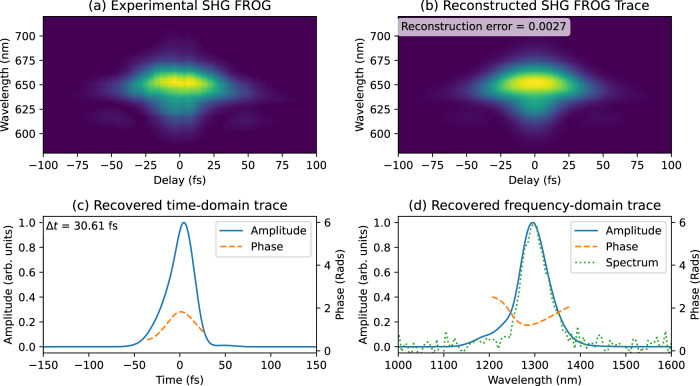
SH-FROG measurements. **a** SH FROG traces experimentally acquired from our AZO film. **b** Reconstructed FROG traces generated by the FROG retrieval algorithm (Retrieval error of 0.0027). **c** Recovered temporal amplitude (solid blue line) and phase (dashed orange line) profiles (FWHM time duration $\triangle t=30.61{fs}$). **d** Recovered spectral amplitude (solid blue line) and phase (dashed orange line) profiles. This plot also reports the experimentally acquired input spectra (dotted green line) for comparison.

**Fig. 4:**
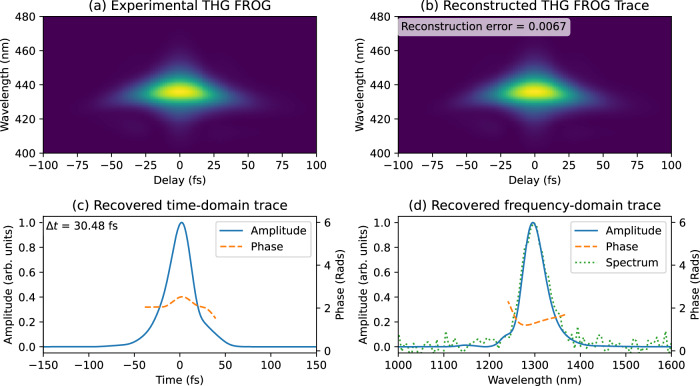
TH-FROG measurements. **a** TH FROG traces experimentally acquired from our AZO film. **b** Reconstructed FROG traces generated by the FROG retrieval algorithm (Retrieval error of 0.0067). **c** Recovered temporal amplitude (solid blue line) and phase (dashed orange line) profiles (FWHM time duration $\triangle t=30.48{fs}$). **d** Recovered spectral amplitude (solid blue line) and phase (dashed orange line) profiles. This plot also reports the experimentally acquired input spectra (dotted green line) for comparison.

Figures 3 and 4 show the results of our SH- and TH- FROG measurements, respectively. Panels (a) report the experimental FROG traces acquired by recording output harmonic spectra as we swept the time delay. Panels (b) are the correspondent FROG traces reconstructed by the retrieval algorithm.

The error in our SH and TH retrievals was 0.0027 and 0.0067, respectively (The number of points considered by the algorithm was 512). These values are well within the bounds accepted by the ultrafast community. Panels (c) show reconstructed time profiles for both amplitude and phase. Finally, in panels (d) we have spectral amplitudes and phases obtained via a Fourier transform of the temporal trace. Additionally, panels (d) report the experimentally acquired input spectra for comparison purposes. All measurements were taken with a 1 kHz laser source providing 30 fs pulses with input energies of 16 μJ for SH and 2 μJ for TH. The temporal intensity profiles as extracted from SH- and TH- FROG are in very close agreement. Retrieved values are also consistent with calibration FROG measurements attained from a standard system employing a bulk BBO crystal (see supplementary material appendix B). Phases recovered by both SH and TH NZI FROG reveal very nearly transform-limited pulses. Our results are also consistent with autocorrelation measurements performed on both SH and TH signals. Retrieved time durations are 30.61 fs for the SH interaction and 30.48fs for TH (both evaluated at FWHM).

### Bandwidth and sensitivity analysis

Understanding how an NZI FROG scheme compares to standard industrial systems (an area that is dominated by SH FROG systems due to their higher sensitivity) will require an examination of key metrics. Notably, the operational bandwidth, sensitivity, time smearing effects, and minimum measurable pulse time duration will be considered.

**Fig. 5:**
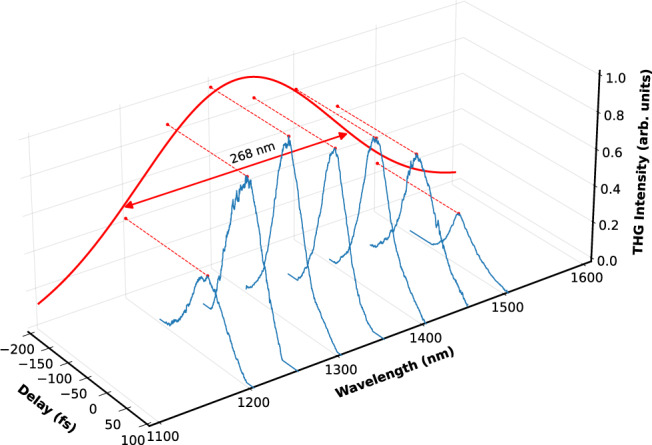
Operational bandwidth measurements. TH autocorrelations as taken at various input central wavelengths. The NZI nonlinear bandwidth has a FWHM of 268 nm (enough to accommodate a 9 fs transform-limited pulse).

In standard FROG systems, the operational wavelength is imposed by the phase-matching constraints as set by the installed bulk nonlinear crystal. Our thin films do not suffer from this limitation as momentum conservation is automatically fulfilled for subwavelength films. While it is true that the phase-matching bandwidth of a bulk nonlinear crystal can be significantly increased by thinning the material down to hundreds of microns, this approach will inevitably lead to a drastic reduction in sensitivity and nonlinear efficiency, along with an increased cost. Instead, the operational wavelength range for our FROG is established by the extent of the NZI region in AZO films. In Fig. 5, we probe this range by recording TH autocorrelations as we tune the central wavelength of the input beam across the NZI window. It is worth mentioning that these measurements have been acquired using a Ti:Sapphire + OPA system, which allowed for a broad tuning of the central wavelength. This source was emitting 100 fs pulses at a repetition rate of 1 kHz and fluence equal to 10 mJ/cm^2^. These results show an operational wavelength range spanning 268 nm centred on 1340 nm, enough nonlinear enhancement bandwidth to accommodate a 9 fs transform-limited pulse. It is also important to note that this nonlinear enhancement primarily stems from the materials NZI nature and not an enhanced χ^(3)^. In fact, AZO’s χ^(3)^ has been shown to be almost flat over the near-IR region^9^.

**Fig. 6:**
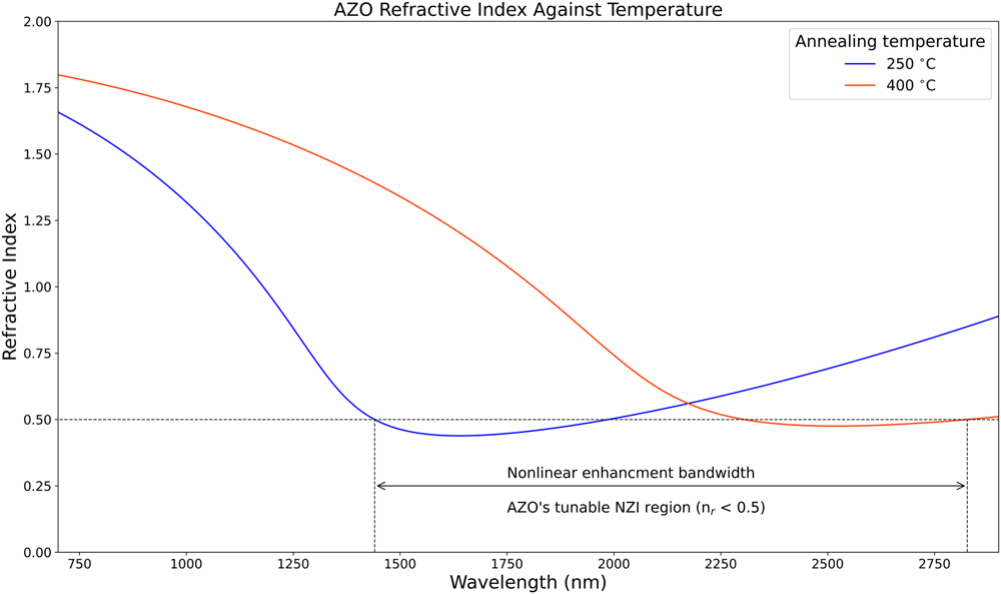
Optical properties engineering via fabrication means. Refractive index dispersions of AZO thin films are reported for two different annealing temperatures (250 °C, blue curve on the left; and 400 °C, red curve on the right). The data in this plot have been repurposed from^50^.

In our measurement, the use of TCO films comes with crucial advantages, namely low fabrication temperature, reduced cost, and static tuneability of the material’s NZI central operational wavelength. Concerning this last point, Fig. 6 reports the possibility to tune the location of the NZI window via fabrication parameters^44^. As we can see, by simply varying growth parameters such as the annealing temperature, the NZI enhancement can be utilised anywhere from 1300 nm to 2800 nm. This range could be extended by acting on film thickness^44^ (see supplementary material appendix C) and to a much larger extent by employing other TCOs (1000–3600 nm)^2^. In this regard, it is important to underline that the fabrication efforts behind the creation of multiple low-cost/low-temp TCO thin films and many bulk crystals opportunely cut for phase matching purposes are substantially different. With the former strategy allowing for a simple and cost-effective way to change the system’s operational wavelength.

**Fig. 7:**
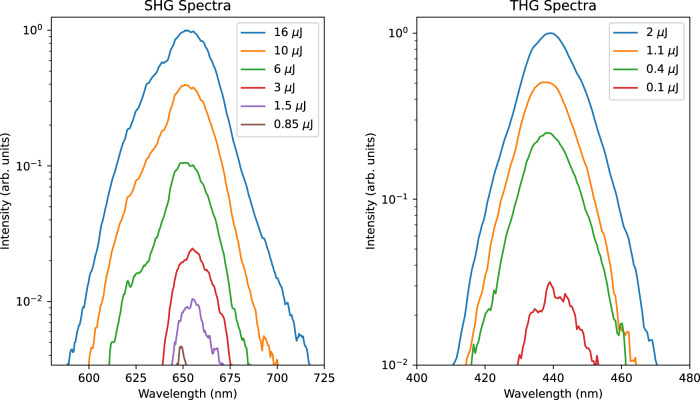
SH and TH sensitivity analysis in NZI films. SH (left) and TH (right) spectra at various input pulse energies from AZO films. All curves are reported at zero delay from the FROG traces. SH plots start at energies as low as 850 nJ, while TH plots start at 100 nJ.

Regarding the sensitivity of a FROG system, this is mainly governed by three fundamental factors: the conversion efficiency of the employed nonlinear crystal, the optical layout, and the technical specification of the spectrometer in use. Commercial modules utilise custom layouts of dispersive elements and photodiode arrays or camera sensors to measure harmonics, which provides optimal collection efficiency and maximises sensitivity. Our FROG measurements were made with a fibre coupled spectrometer and applied to optical pulses with energies as low as 100 nJ for the TH-FROG and 850 nJ for SH-FROG, all at a 1 kHz repetition rate. This can be seen in Fig. 7 which shows SH and TH spectra at various pulse energies at zero delay (Additional plots showing SH and TH signal vs power scaling can be found in supplementary material Appendix D). These remarkable sensitivity values could be further improved by simple technical expedients (e.g., replacing fibre-coupled integrated spectrometers with free space coupled systems).

## Discussion

It is worth underlying that here we are fundamentally comparing our system’s performance against those of SH-generation-based devices. In other words, we are comparing the TH conversion efficiency from ultra-thin elements with the SH conversion efficiency from bulk crystals, where the latter is conventionally several orders of magnitude larger than the former. These considerations further clarify the importance of comparing our system’s TH conversion efficiency to other ultra-thin, out-of-plane configurations, where the intrinsic advantages of employing NZI materials can be clearly understood.

The final assessment to be considered is the minimum measurable pulse duration. Commercial systems utilise parametric nonlinearities, which can be considered instantaneous, and thus the maximum bandwidth is instead set by phase-matching considerations. In more complex geometries, such as those adopted in single-shot FROGs, the pixel density on the sensor and the geometrical distortions of the wavefront also play an important role in determining the maximum pulse bandwidth.

While these bandwidth limitations are general to almost any FROG system, they do not apply to our NZI FROG, which is intrinsically phase-matched and operates on ultra-thin films. Regarding this last point, it is worth noting that our module is also unaffected by the issue of longitudinal geometrical distortion (time-smearing), which directly steams from propagating in a thick medium^32^. In fact, relaxed phase-matching conditions allow for operating at almost arbitrary angles between the two interacting beams, while the subwavelength thickness of our films minimises longitudinal distortion. It is also instrumental to highlight that the absence of phase-matching has the additional practical benefit of drastically relaxing the alignment procedure and simplifying the overall system design.

Regarding the “speed” of the excited nonlinearities, we are certainly operating in a regime where the slow nonlinearities do not contribute. This is naturally demonstrated by the efficiency of the THG process, for which the polarisation term must oscillate at the same frequency as the third harmonic and is thus instantaneous ($\approx$1 fs). Furthermore, the nonlinear bandwidth does not limit the shortest pulse duration measurable with a FROG system^45^. As a consequence, despite measured pulses having a time duration of about 30 fs, it is reasonable to assume that our measuring capability could handle much shorter pulses.

A conventional FROG scheme, as was constructed in this study, is more than adequate as a proof-of-concept. Still, several advancements could be implemented. For instance, single-shot FROG is a well-known configuration that can record an entire FROG scan with a single pulse. If AZO were utilised for this configuration, all previously discussed advantages stemming from the operation in the NZI region would still be valid. The sensitivity would be improved dramatically over our current system due to the use of free space light collection instead of a fibre coupled spectrometer. There is also the issue of longitudinal geometrical distortion, which occurs in single-shot FROG schemes due to delay variation along the direction of the signal beam. Unlike the bulk crystal counterparts, this issue would be minimised with thin-film geometry, improving the accuracy further.

Our system also benefits from sub-micron propagation distances (so that there is no need for dispersion compensation) and CMOS compatibility. From this last point, it could be possible to devise a fully integrated single shot FROG^46,47^ which can also exploit dual harmonic generation. While it may be possible to think of such a system in a modular fashion (placing integrated components in series to mimic the classic design of a single-shot FROG), it would be more effective to design the entire device from the ground up by optimising the performance of the whole unit, while maximising compactness.

Recently machine-learning based approaches have been successfully employed to retrieve the amplitude and phase of pulses from FROG traces without the need to apply the iterative FROG retrieval algorithm, significantly hastening the characterisation process^48^. An ongoing analysis, that will be discussed in greater detail in a follow-up publication, hints to an increased retrieval accuracy when the machine learning algorithm utilises both SH and TH traces, which are uniquely available to our system. Having access to both SH and TH improves not only the resilience and robustness of measurements, but also directly solves the direction-of-time ambiguity inherent to SH FROG traces. It also brings in the ease and flexible range of the needed measurements. For example, if the SH wavelength becomes too large for available spectrometers, we can instead record the TH, and the same is true of the reversed situation^49^.

In summary, in the quest to find the “killer” application for NZI nonlinear systems, we have successfully designed, assembled, and characterised a proof-of-concept FROG scheme with a highly nonlinear AZO ultra-thin film at its core. From a practical point of view, this stands to be the first application to match, and in many key metrics, surpass state-of-the-art commercial products. From a more fundamental perspective, our results show that NZI materials should be the number one choice whenever ultra-short nonlinear propagation distances are required and/or out-of-plane geometry are used. Through the employment of TCOs and their unique NZI properties, our scheme allows for efficient, simultaneous, and naturally phase-matched SH- and TH-generation. In turn, this leads to drastically relaxed alignment conditions, extended operational bandwidth, and unprecedented robustness and flexibility in the data analysis. The high TH conversion efficiency coupled with the ability to generate a surface SH simultaneously allows the measurement of signals across a wide spectral range with just a single spectrometer. The CMOS compatibility and ease of fabrication of TCOs nano-layers can potentially enable fully integrated single-shot configurations, which can also benefit from dual-harmonic machine learning algorithms for ultra-fast pulse retrieval.

## Methods

### Near-zero-index frequency resolved optical gating setup

A 35 mW beam of 100 fs pulses at 1 kHz is generated by an OPA (wavelength tuneability between 1100 nm and 1600 nm) pumped with a Spectra-Physics Spitfire chirped pulse amplification system was used to perform the measurements reported in Figs. 2 and 5. The FROG traces in Figs. 3 and 4 and the spectra in Fig. 7 were recorded with the AZO-based FROG scheme depicted in Fig. 1. This scheme was supplied 30 fs pulses at 1 kHz by a Light conversion TOPAS HE optical parametric amplifier, pumped by a Legend Elite laser (Coherent) emitting 30 fs pulses at 8 mJ. The beam is then directed onto a split metal mirror with one half attached to a fine translation stage (allowing delay control), and then through two arms. Both beams are focused onto a 270 nm AZO film via a spherical mirror with radius of curvature 250 mm, resulting in a focal spot diameter of 121 μm (full width at 1/e^2^ point measured via Basler IR Camera) and pulse energy ranging up to 16 μJ/cm^2^ at the film’s surface. The incident angle of the beam onto the film is very close to normal incidence. An additional beam line, accessible via a flip mirror (see Fig. 1 for reference), images the focal plane of the beam’s intensity profile onto the IR camera (see supplementary material appendix E). This allowed us to attain a maximum spatial overlap among the two arms in the focal plane, a direct measure of the used fluence, and also an accurate temporal overlap by looking at the interference pattern. A fibre coupled spectrometer calibrated in power spectral density collects the generated SH and TH. The AZO sample are manufactured via pulsed laser deposition in an oxygen-deprived environment^24^. To attain direct SH and TH autocorrelation traces in Fig. 5, we modify the harmonic collection system, swapping the spectrometer for a Si-photodiode with an optimal spectral response in the visible.

### Angular nonlinear signal optimisation

We also investigated the TH conversion efficiency of our AZO thin film at various incident angles. The input power was monitored by directing a pulse onto a 90/10 beam-splitter and measuring the smaller fraction with a photodiode. The larger fraction of power is focused onto the AZO film via a 100 mm plano-convex lens, resulting in a spot size of 80 μm. The generated TH is then collected with a Si photodiode for incident beam angles of 0°, 30° and 60°.

### Measurement retrieval algorithm

Standard FEMSOFT algorithms for SH and TH FROG methods were used to recover the traces. This algorithm dynamically switches between methods, with a mixture of iterative and stochastic approaches utilised to increase the rate of convergence. The FROG algorithm has a fundamental resistance to noise due to the numerous pulses required to form any single FROG trace.

## Supplementary information

Supplementary Information

## Data Availability

All data generated in this study have been deposited in the Heriot–Watt University database under accession code 10.17861/8bca228b-a6d0-4afa-a390-d1e2a089c4aa.
