# Supplementary material for: Near-zero-index ultra-fast pulse characterization
Source: Nat Commun. 2022 Jun 20;13:3536. doi: 10.1038/s41467-022-31151-4 (PMC9209551; doi:10.1038/s41467-022-31151-4)
Supplement: Supplementary file 1 — Supplementary Information [file 41467_2022_31151_MOESM1_ESM.pdf]

Supplementary Material

Near-zero-index ultra-fast pulse characterization

Wallace Jaffray[1], Federico Belli[1], Enrico Carnemolla[1], Catalina Dobas[1], Mark Mackenzie[1], John Travers[1], Ajoy K. Kar[1], Matteo Clerici[2], Clayton DeVault[3], Vladimir M. Shalaev[4], Alexandra Boltasseva[4], and Marcello Ferrera[1]

[1]Institute of Photonics and Quantum Sciences, Heriot-Watt University, SUPA, Edinburgh, EH14 4AS, UK
[2] James Watt School of Engineering, University of Glasgow, Glasgow G12 8QQ, UK
[3] School of Engineering and Applied Sciences, Harvard University, Cambridge, MA 02138, USA
[4] School of Electrical and Computer Engineering and Birck Nanotechnology Center, Purdue University, West Lafayette, Indiana 47907, USA
**Corresponding author: m.ferrera@hw.ac.uk*

Appendix A

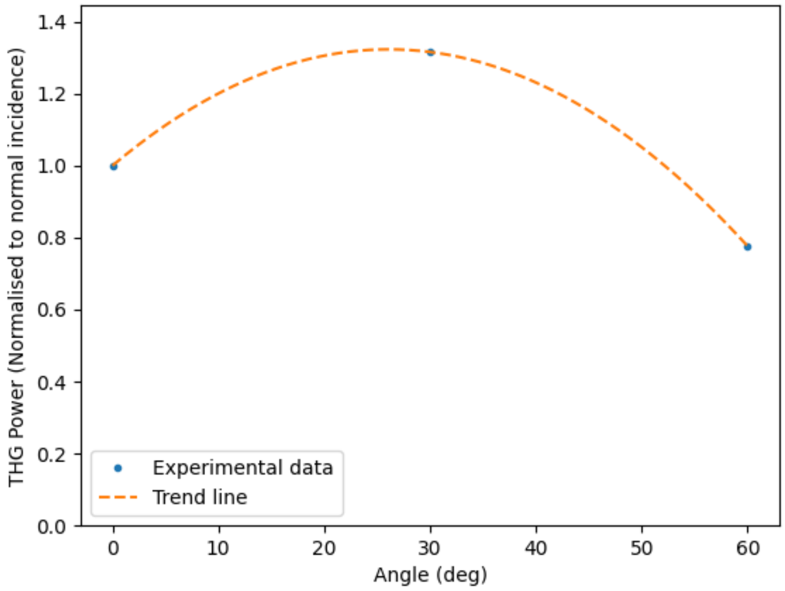

Fig. 1: Angular dependence of THG for a single beam with horizontal polarisation. 6 µJ pulses with FWHM of 100 fs were used. Input and output pulse energy was monitored on a pulse-by-pulse basis to ensure accuracy.

The angular dependence of THG generation was characterised with a beam of 100 fs pulses at a constant energy of 6 µJ. This measurement was completed in order to ascertain if the input angle of our FROG system could have a bearing on sensitivity due to NZI angular enhancement. It is important to note that the trend in this measurement cannot be completely ascribed to NZI angular enhancement as we are measuring external efficiency and the transmission into the film is included in this measurement.

Appendix B

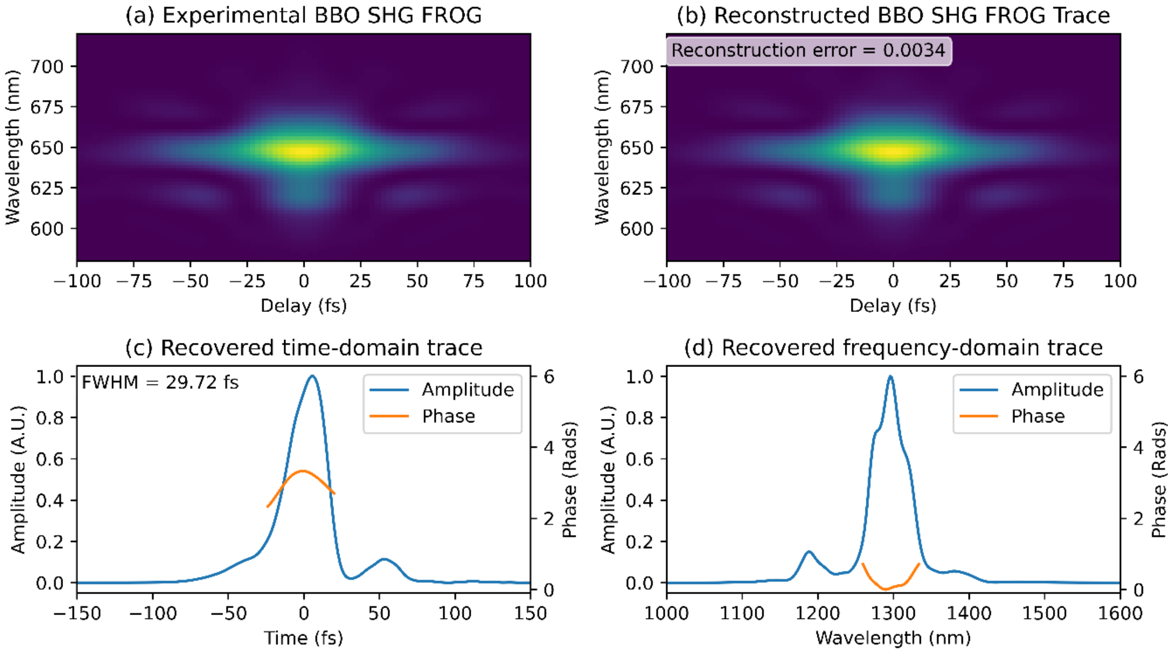

Fig. 2: BBO crystal SH-FROG measurements. **(a)** SH FROG traces experimentally acquired from a BBO crystal. **(b)** Reconstructed FROG generated by the FROG retrieval algorithm (Retrieval error of 0.0034). **(c)** Recovered temporal phase and amplitude profile (FWHM time duration of 29.72 fs). **(d)** Recovered spectral phase and amplitude profile.

Figure 2 above show the results of our BBO crystal calibration test. Panel a) report the FROG traces acquired by recording the SH spectra generated by a BBO crystal as the time delay is varied. Panel b) gives correspondent reconstructed FROG traces provided by a standard retrieval algorithm. The average error this retrieval is 0.0034 which is within bounds defined in our retrieval software's operating manual for SH traces. Panels c) show reconstructed time profiles for both amplitude and phase and, finally, in panels d) we have spectral amplitudes and phases as found via a Fourier transform on the temporal trace. This measurement was taken with a 1 kHz laser source providing 30 fs pulses.

Appendix C

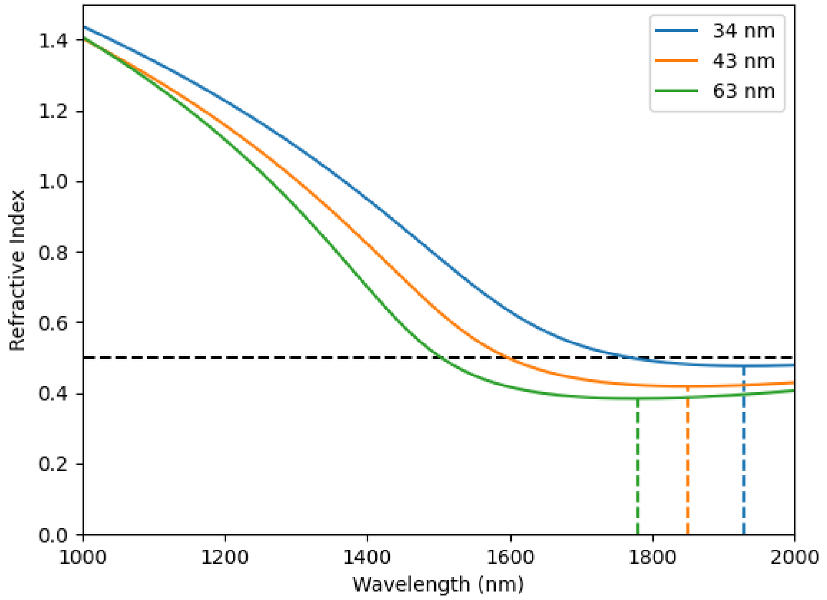

Fig. 3: AZO's refractive index tunability via sample thickness. The black dashed line indicates refractive index of 0.5. Further tunability in ENZ point could be achieved by varying the annealing temperature or using other TCO such as ITO. Data in this plot was taken from preliminary investigations into AZO's tunability.

The above figure demonstrates how further tunability of the NZI range can be achieved by varying sample thickness. Three thickness were considered, 34 nm, 43 nm, and 63 nm, all of which were annealed at the same temperature. The black dashed line indicates where the real refractive index drops below 0.5 and NZI properties begin to emerge. The data for this figure comes from preliminary investigation performed at Purdue university where we are investigating the extent of AZO's tunability range. It is also worth mentioning that this thickness dependence has been previously investigated in [45] and the results were found to be comparable.

Appendix D

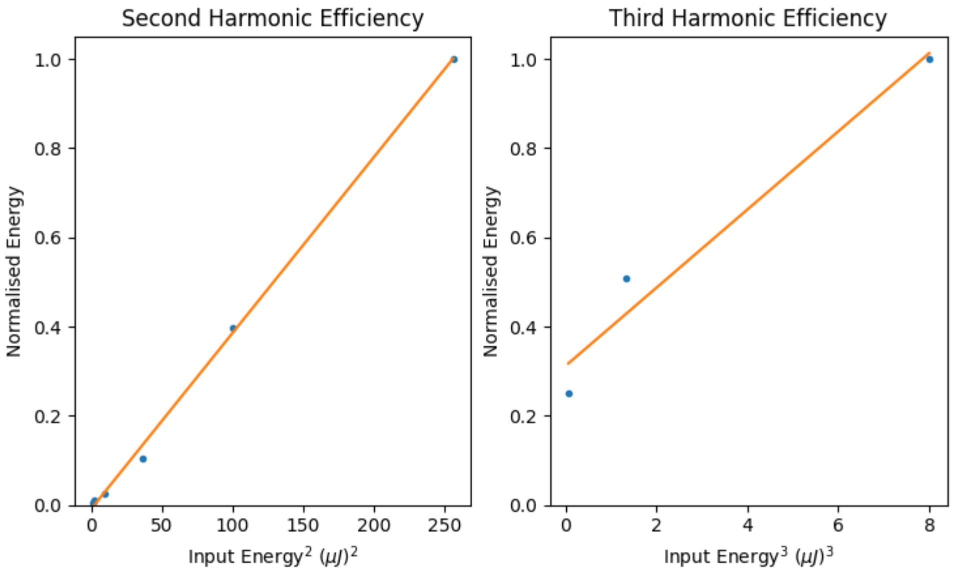

Fig. 4: Second and third harmonic generation power scaling of a 270 nm AZO film. Measurements were made with 30 fs pulses at a 1 kHz repetition rate and 1300 nm.

Figure 4 above shows second and third harmonic power scaling. The x-axis on each figure has been scaled appropriately such that a linear fit can be applied to each nonlinear process. Second harmonic generation shows a quadratic dependence on input energy whilst third harmonic generation shows a cubic dependence which is to be expected. All measurements were made with a 1kHz laser producing 30 fs pulses at 1300 nm.

Appendix E

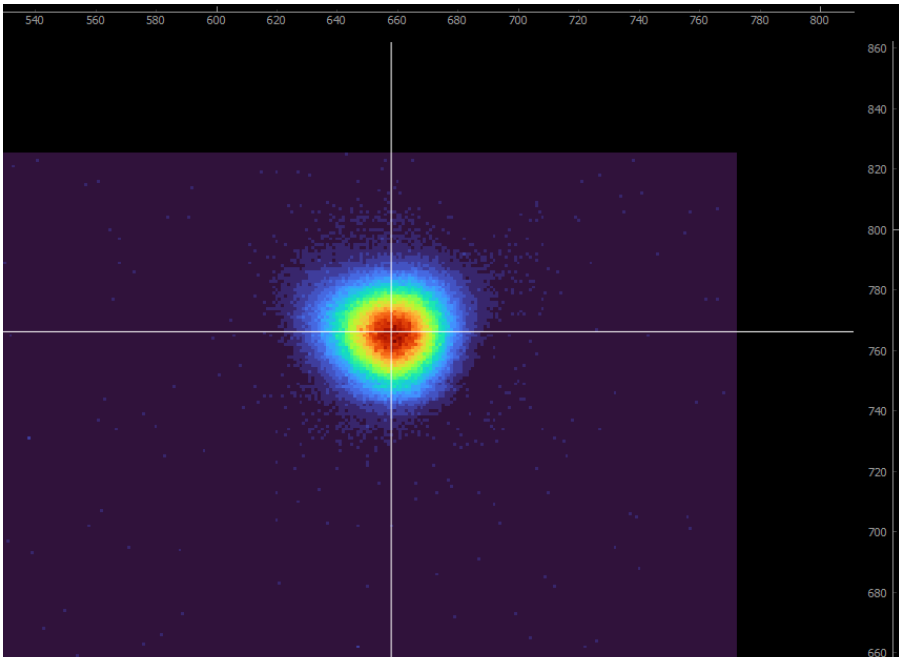

Fig. 5: Beam profile measured via camera at focal plane. Beam waist found to be 121 μm.

The beam waist at the air-AZO interface was measured by picking off the beam with a mirror and directing it towards a Basler IR Camera. The same camera was used to overlap the two beams temporally as interference fringes could be observed between the two pulses as the delay was tuned.
